# Orthodontics‐Assisted Autogenous Tooth Transplantation for Maxillary Incisor Injury in Pediatric Patient With Crowding and Class II Malocclusion: A Case Report

**DOI:** 10.1002/cre2.70405

**Published:** 2026-07-08

**Authors:** Nga Thi Nguyen, Hanh Thi‐My Tran, Nga Minh Truong

**Affiliations:** ^1^ Hanoi Medical University Thanh Hoa Campus Thanh Hoa Vietnam; ^2^ Nu Cuoi Dental Clinic Thanh Hoa Vietnam; ^3^ Department of Pediatric Dentistry, School of Odonto‐Stomatology Hanoi Medical University Ha Noi Vietnam; ^4^ Faculty of Dentistry University of Medicine and Pharmacy at Ho Chi Minh City Ho Chi Minh City Vietnam

**Keywords:** autogenous tooth transplantation, crowding and class II malocclusion, maxillary incisor injury, orthodontic treatment, pediatric patient, post‐endodontic periapical complications, traumatic dental injury

## Abstract

**Objectives:**

This case report describes orthodontics‐assisted autogenous tooth transplantation for a maxillary incisor injury in a pediatric patient with crowding and Class II malocclusion.

**Material and Methods:**

A 10‐year‐old boy presented with a compromised maxillary left central incisor with a periapical lesion after failed endodontic treatment, together with bimaxillary crowding and Class II malocclusion. The mandibular right second premolar was transplanted to replace the maxillary incisor and was integrated with comprehensive orthodontic treatment.

**Results:**

After transplantation, endodontic treatment was completed when pulp vitality could not be maintained. During long‐term follow‐up, the transplanted tooth remained clinically stable, with healthy periodontal tissues and no progressive root resorption or bone loss. Orthodontic treatment achieved stable occlusion, improved function, and satisfactory smile esthetics.

**Conclusions:**

Orthodontics‐assisted autogenous tooth transplantation may be a biologically favorable option for replacing a traumatized maxillary incisor in growing patients with crowding and malocclusion. Careful donor selection, coordinated orthodontic timing, and long‐term follow‐up are essential for stable functional and esthetic outcomes.

**Abbreviations**
ANB: A point‐Nasion‐B point angle
DDS: Doctor of Dental Surgery
FMA: Frankfort‐mandibular plane angle
IMPA: incisor mandibular plane angle
L1‐NB: lower incisor to Nasion‐B line (angular/linear measurement)
MTA: mineral trioxide aggregate
SNA: Sella‐Nasion‐A point angle
SNB: Sella‐Nasion‐B point angle
U1‐NA: upper incisor to Nasion‐A line (angular/linear measurement)

## 1 Introduction

Maxillary incisor injuries in children and adolescents are a common clinical problem, representing the majority of all dental trauma cases (El‐Kenany et al. 2016). The most frequently encountered injuries include crown fractures, pulp involvement, and periodontal tissue damage, in which extensive crown fractures associated with pulpal injury increase the risk of complications such as internal resorption and alveolar bone loss if not managed appropriately (Bastone et al. 2000; Güngör 2014). Restoring a missing permanent incisor in patients who have not yet completed craniofacial growth remains a major clinical challenge (Duggal et al. 2017). Traditional treatment modalities such as fixed dental bridges require preparation of sound tooth structure, compromise the alveolar bone, and may result in long‐term damage (Palmer 2010). Implant placement is also limited in this age group, as ongoing skeletal and facial development poses a high risk of implant infra‐occlusion or axial displacement over time (Lekholm 2003).

In this context, tooth autotransplantation has emerged as a feasible and biologically favorable treatment option (Güngör 2014; Park et al. 2010). This technique involves transplanting the patient's own tooth to replace the missing one, offering key advantages such as preservation of a vital periodontal ligament, maintenance of alveolar bone height, facilitation of physiologic craniofacial development, and compatibility with adjunctive orthodontic treatment (Park et al. 2010; Andreasen et al. 1990; Almpani et al. 2015; Tsukiboshi 2002). Studies have demonstrated high success rates of autotransplantation, particularly in children and adolescents, owing to their superior healing capacity (Andreasen et al. 1990; Czochrowska et al. 2002; Kafourou et al. 2017). Long‐term investigations have reported survival and clinical success rates exceeding 80% (Czochrowska et al. 2002; Montesinos et al. 2025). The success of the procedure depends on multiple factors, among which the most critical are the stage of root development, the difficulty of extraction, and the condition of the alveolar socket at the recipient site (Kafourou et al. 2017). In addition to replacing the missing tooth, patients in the growth phase often present with malocclusions. In cases with Class II skeletal discrepancies accompanied by proclined incisors and severe crowding, orthodontic treatment is essential to restore dentoalveolar harmony. Integrating orthodontic therapy with autotransplantation facilitates space closure, enables controlled force application on the transplanted tooth, and optimizes overall esthetic and functional outcomes of the dental arches (Brierley et al. 2017).

Based on prior literature, the use of premolar autotransplantation to replace maxillary incisors has been reported to yield favorable esthetic and functional outcomes (Suwanapong et al. 2021; Piroozmand et al. 2018). The present case describes a 10‐year‐old male patient with a maxillary left central incisor presenting with a periapical lesion following failed endodontic treatment, accompanied by bimaxillary crowding and Class II malocclusion. The patient was managed with autotransplantation of the mandibular right second premolar to replace the missing incisor, combined with comprehensive orthodontic treatment. The treatment process, associated challenges, and clinical outcomes over a 7‐year treatment and follow‐up period are presented in detail to provide additional evidence supporting the long‐term effectiveness of autogenous tooth transplantation combined with orthodontic therapy in managing complex cases in growing patients.

## 2 Case Presentation

### 2.1 Patient Information

A 10‐year‐old male patient presented to the Nu Cuoi Dental Clinic on June 1, 2018, with pain and swelling in the maxillary anterior region, accompanied by esthetic concerns due to discoloration, two‐thirds crown fracture, and mobility of the maxillary left central incisor (tooth 21). The patient frequently complained of pain and felt self‐conscious about smiling during social interactions. In addition, the family had noticed protrusive incisors and sought treatment to improve facial and dental esthetics. The parents expressed the desire for a treatment solution that would restore both masticatory function and esthetics.

Two years earlier, the patient sustained dental trauma while playing football, resulting in fractures of the anterior teeth and abrasions of the upper lip. Tooth 21 was diagnosed with an enamel‐dentin‐pulp fracture and subsequently underwent endodontic treatment at a private clinic. Pulp extirpation and root canal obturation were performed over two visits; however, the tooth was never permanently restored. Persistent dull pain, progressive gray discoloration, and redness and swelling of the labial gingiva developed within the following weeks. Approximately 3 months later, a small sinus tract appeared on the gingiva of tooth 21 and intermittently discharged purulent fluid. Each recurrence was managed with oral antibiotics, which provided only temporary relief, with symptoms reappearing every few months. In addition, the upper lip had sustained a minor laceration during the fall, which was sutured and healed without complications. No other teeth were affected. The medical history was unremarkable; the patient was healthy with no known allergies or systemic conditions.

### 2.2 Clinical Findings and Diagnostic Assessment

At the initial visit to our clinic, the family expressed that their primary concern was the child's esthetics and psychological well‐being. Following the traumatic event, the patient had smiled infrequently, tended to avoid social interactions, and exhibited signs of shyness and reduced self‐esteem.

Extraoral examination: Facial proportions were symmetrical and harmonious, with no evidence of craniofacial deformity. However, smile esthetics were noticeably compromised due to discoloration and signs of infection associated with the maxillary left central incisor. The patient appeared reluctant to smile, reflecting the psychological impact of the previous trauma (Figure 1).

**Figure 1:**
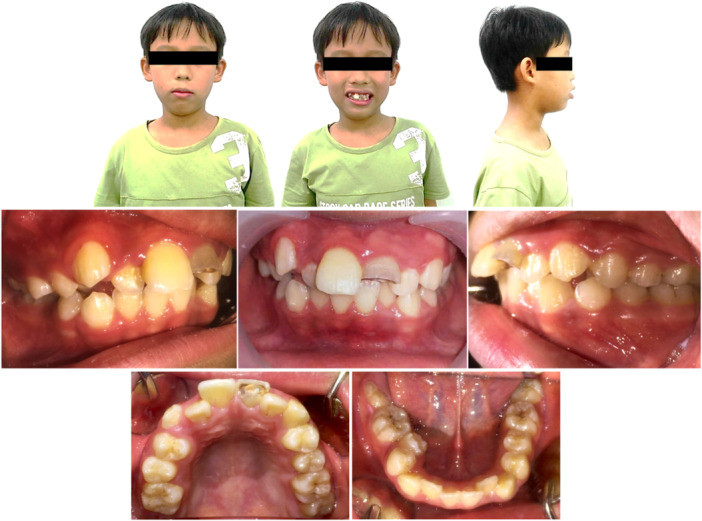
Extraoral and intraoral photographs of the patient before treatment.

Intraoral examination: The maxillary left central incisor was grayish in color, exhibited Grade II mobility, and presented with a small sinus tract on the labial gingiva, accompanied by mild swelling and tenderness on palpation. The remaining maxillary incisors had normal morphology, position, and color. Additionally, the mandibular left first premolar was positioned buccally, resulting in mild crowding in the premolar region and altered tooth angulation. Both arches demonstrated moderate irregularity (Figure 1).

Subsequently, the patient underwent panoramic and lateral cephalometric radiography at the start of treatment. The radiographic images revealed a periapical lesion associated with tooth 21, dental crowding, and maxillomandibular discrepancy (Figure 2).

**Figure 2:**
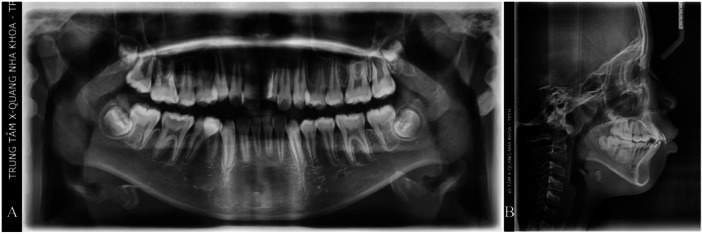
Pretreatment radiographic images. (A) Panoramic radiograph showing periapical bone loss associated with tooth 21, along with a straight, well‐formed root of tooth 45 suitable as a donor candidate for autotransplantation. (B) Lateral cephalometric radiograph.

A detailed cephalometric analysis revealed that the anteroposterior position of the maxilla remained within normal limits, whereas the mandible was markedly underdeveloped. Specifically, SNA was 77.21°, falling within the reference range, while SNB was 71.47°, significantly below normal, resulting in an increased ANB angle of 5.74° (> 4°). This confirms a skeletal Class II relationship, primarily attributed to mandibular retrusion on a nearly normal maxillary base. Additionally, both the Björk sum (404.94°) and FMA (33.94°) were markedly elevated relative to normative values, indicating a vertical growth pattern with downward and backward rotation of the mandible. This pattern contributes to a retrognathic chin appearance and further exacerbates the Class II malocclusion. On this skeletal foundation, the incisor positions showed compensatory but esthetically unfavorable inclinations. The maxillary incisors were proclined and protrusive, with U1‐NA (°) = 28.37° (higher than the normative value of approximately 22°) and U1‐NA (mm) = 6.24 mm (greater than the typical 4 mm), indicating marked proclination and protrusion of the upper incisors. To achieve occlusal contact with the maxillary dentition, the mandibular incisors were also excessively proclined, demonstrated by L1‐NB (°) = 33.13° and L1‐NB (mm) = 8.50 mm, and an IMPA of 96.73°, confirming pronounced forward inclination relative to the mandibular plane. The combined protrusion of both upper and lower incisors resulted in a reduced interincisal angle of 112.75° (substantially lower than the reference value), indicating bimaxillary incisor proclination, often associated with lip protrusion and a convex facial profile. The overjet was recorded at 5.59 mm, while the overbite was 2.13 mm, the latter remaining within normal limits.

Based on these findings, the patient exhibited skeletal Class II due to mandibular retrusion on a vertical growth pattern. The malocclusion represented a combination of skeletal discrepancy and dental compensation, with excessive incisor proclination in both arches and crowding measuring approximately 6 mm in the maxilla and 8 mm in the mandible, contributing to a bimaxillary dentoalveolar protrusion on a Class II skeletal base. This condition had a significant impact on facial esthetics and occlusal function. Therefore, an extraction approach was indicated to achieve dual objectives: resolving crowding and creating space for incisor retraction, thereby reestablishing both esthetic and functional harmony.

### 2.3 Therapeutic Intervention

After a multidisciplinary consultation involving orthodontics, restorative dentistry, and periodontics, a comprehensive treatment plan was established. The maxillary left central incisor, which had undergone endodontic treatment but failed, presented with periapical bone loss and a sinus tract; therefore, extraction was mandatory. At the same time, to address bimaxillary crowding (6 mm of space deficiency in the maxilla and 8 mm in the mandible) and the dental Class II relationship caused by dentoalveolar protrusion, extraction of four premolars (teeth 14, 24, 34, and 45) was proposed. Among these, tooth 45—which had a straight root, adequate length, and intact morphology—was selected as the donor tooth for autotransplantation to replace tooth 21. Preoperative radiographic assessment and intraoperative observations showed that tooth 45 had nearly completed root development with relatively mature root walls, while the apical foramen remained open but with a relatively small diameter, corresponding approximately to stage IV of Cvek's classification of root development (Cvek 1992). This morphology indicated that the donor tooth was close to root maturation rather than being in the earlier stage of incomplete root formation typically considered most favorable for pulp revascularization after transplantation. Furthermore, because tooth 45 was positioned lingually, retaining it would not be favorable for orthodontic alignment. All alternative treatment options were thoroughly discussed with the patient and family. After counseling and careful consideration of the benefits, risks, and treatment sequence, the clinicians and family agreed to proceed with the extraction and autotransplantation of tooth 45 into the site of tooth 21, combined with comprehensive orthodontic therapy.

Treatment began with bonding fixed appliances on both arches. In the maxillary arch, teeth 14 and 24 were extracted first to create space for the alignment and repositioning of teeth 11 and 21. Alignment continued until a sufficiently rigid 16 × 22 Nickel‐Titanium archwire could be placed to serve as a stable splint for the transplanted tooth. After 4 months of orthodontic leveling, the conditions were deemed adequate for transplantation surgery. Under local anesthesia, tooth 21 was atraumatically extracted, the periapical inflammatory tissue was curetted, and the recipient socket was prepared to match the dimensions of tooth 45. Tooth 45 was carefully extracted using surgical forceps with minimal trauma to preserve periodontal ligament viability. The donor tooth was then immediately transplanted into the prepared socket at the 21 position, with adjustments to its angulation and cervical height to harmonize with the arch. A slight reduction on the labial surface was performed to compensate for torque differences between a premolar and the bracket base of a maxillary incisor, allowing the tooth to fit correctly into the passive archwire without applying orthodontic force. In this case, the transplanted tooth was bonded with a bracket immediately after surgery, providing initial stabilization and enabling early orthodontic activation. Occlusal interferences on the transplanted tooth were checked and eliminated in centric and functional movements (Figure 3). The patient was instructed to maintain a soft diet for 2 weeks, practice meticulous oral hygiene, and attend regular follow‐up appointments to monitor healing. Pulp vitality was evaluated at 2, 4, and 6 weeks. At 8 weeks, the tooth showed no response to pulp sensibility tests; therefore, root canal treatment was performed after sufficient stabilization to ensure long‐term prognosis (Figure 4).

**Figure 3:**
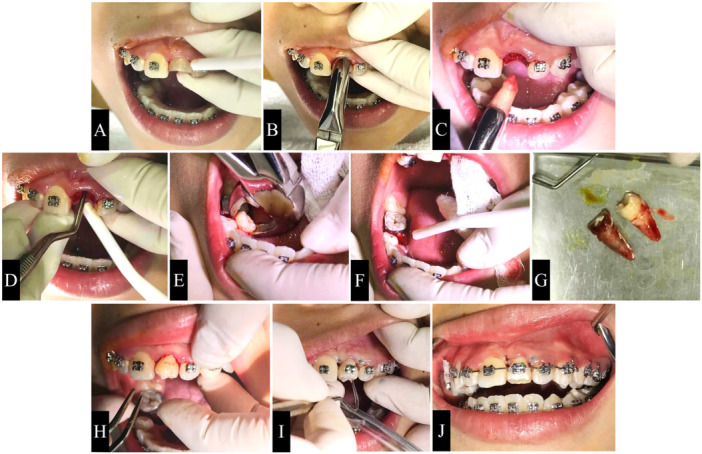
Step‐by‐step procedure of the autotransplantation. (A) Pre‐extraction view of tooth 21 in the dental arch. (B–D) Gentle extraction of tooth 21, debridement of periapical inflammatory tissue, and preparation of the recipient socket. (E, F) Atraumatic extraction of tooth 45 using surgical forceps and cleansing of the surgical site. (G) Tooth 45 (right) and tooth 21 (left) after extraction and before transplantation, showing comparable straight root lengths. (H) Transplantation of tooth 45 into the 21 site with adjustment of root angulation and cervical height. (I, J) Completion of the transplantation by bonding a fixed orthodontic bracket and suturing, with labial reduction of the transplanted tooth to adapt to the bracket base of tooth 21, ensuring passive positioning on the archwire and elimination of occlusal contacts in centric and functional movements.

**Figure 4:**
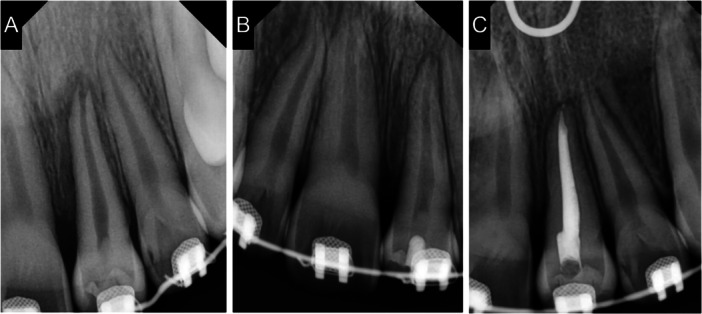
Sequential radiographs of the transplanted tooth (tooth 45 replacing tooth 21). (A) At 1 week, the transplanted tooth shows an open apex immediately after transplantation. (B) At 6 weeks, the transplanted tooth shows no response to pulp sensibility testing; endodontic access was initiated, and calcium hydroxide was placed as an intracanal medicament. (C) At 12 weeks, definitive endodontic treatment was performed on the transplanted tooth.

The mandibular right second premolar (referred to as the “transplanted tooth”) was initially left without endodontic treatment, with the aim of preserving pulp vitality to allow continued root development, as the apex remained open. At the 1‐week follow‐up, the transplanted tooth exhibited no spontaneous pain and no sensitivity to percussion or palpation, adjacent teeth responded normally to cold and electric pulp testing. Radiographic examination showed an open apex with a uniform periodontal ligament space and no periapical radiolucency, therefore, vitality preservation was continued.

At the 6‐week follow‐up, the transplanted tooth showed no response to pulp sensibility tests. Radiographs revealed an incompletely closed apex with slight periapical radiolucency, suggesting a reduced likelihood of maintaining pulp vitality. Endodontic access was therefore initiated, and calcium hydroxide was placed as an intracanal medicament to promote disinfection and support further apical closure. At the 12‐week follow‐up, the patient remained asymptomatic, and radiographs demonstrated sufficient apical closure to allow conventional obturation. Based on these findings, definitive endodontic treatment was completed. After rubber dam isolation, the canal was accessed, shaped, and irrigated with 2.5% sodium hypochlorite. The root canal was obturated using gutta‐percha in combination with an MTA‐based sealer (MTA Fillapex, Angelus, Brazil). The pulp chamber was restored with light‐cured composite resin (Filtek Z250, 3M ESPE, St. Paul, MN), followed by occlusal adjustment as needed.

## 3 Follow‐up and Outcomes

Clinical and radiographic outcomes were evaluated according to previously reported criteria for transplantation success, including the absence of pain or pathologic mobility, probing depths ≤ 3 mm, preservation of periodontal ligament space on radiographs, and the absence of progressive root resorption.

During the follow‐up visits within the first 3 months after autotransplantation, radiographs showed progressive stabilization of the bone surrounding the transplanted root, with evidence of new bone formation around the apex. During this period, orthodontic forces were applied only to other areas of the arch, while the transplanted tooth was protected from direct loading.

Three months after surgery, once radiographs confirmed new bone formation around the transplanted root, orthodontic forces were initiated in the maxillary anterior region. At subsequent annual follow‐up appointments through June 2025, the patient maintained good oral hygiene and reported no pain or discomfort. The transplanted tooth exhibited physiologic mobility, no tenderness to percussion or palpation, and stable probing depths of ≤ 3 mm. Periapical radiographs demonstrated healthy periodontal and alveolar bone healing around the transplanted root, with no signs of root resorption or progressive bone loss. Adjacent teeth and both dental arches remained stable without pathological changes. Comprehensive orthodontic treatment was carried out from 2018 to December 2019, including space closure after premolar extractions, alignment of both arches, and establishment of a Class I occlusal relationship. The final results showed well‐aligned arches, symmetrical bilateral occlusion, and ideal incisor angulation on post‐treatment radiographs. The transplanted tooth remained stable, with healthy peri‐gingival tissues, successful endodontic treatment, and a composite build‐up harmonized with the morphology of tooth 11, ensuring optimal esthetics.

The treatment process began in July 2018, was completed in September 2020, and continued to be monitored through June 2025. During the follow‐up evaluations in years 3, 4, and 5, the patient underwent periodic assessments to refine the occlusion, and the autotransplanted tooth replacing the maxillary left central incisor responded well to orthodontic forces. Throughout all follow‐up visits, up to 5 years post‐transplantation, the patient exhibited no clinical symptoms involving the maxillary or mandibular incisors or the adjacent teeth. Clinical examinations consistently remained within normal limits, with healthy periodontal tissues and no pathological findings on radiographs. The extraction space was completely closed during orthodontic treatment, along with substantial improvements in masticatory function and smile esthetics (Figures 5 and 6).

**Figure 5:**
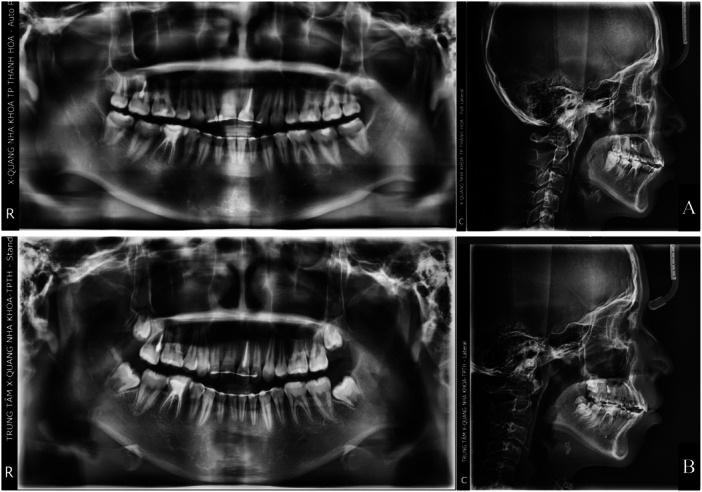
Post‐treatment and follow‐up radiographs. (A) At the end of treatment (after 2 years). (B) At the 5‐year follow‐up.

**Figure 6:**
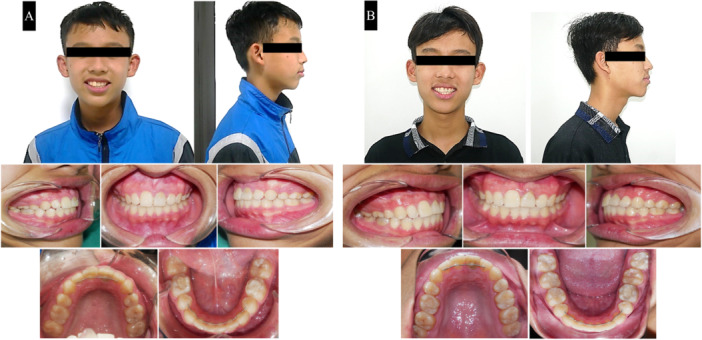
Intraoral and extraoral photographs of the patient after treatment and during follow‐up. (A) At the end of treatment (after 2 years). (B) At the 5‐year follow‐up.

## 4 Discussion and Conclusions

Traumatic injury to incisors is a common condition in adolescents and may lead to various complications, among which root resorption is a frequent late‐onset event that progresses slowly and silently. Therefore, periodic clinical and radiographic follow‐up is essential for early detection and timely management (Galler et al. 2021). The loss of a permanent incisor in a growing patient presents a major therapeutic challenge because early implant placement is contraindicated, and fixed prosthetic options may negatively affect adjacent teeth. In this context, autogenous tooth transplantation has emerged as a valuable treatment option, providing both functional restoration and long‐term esthetic benefits (Chalissery et al. 2016). In this report, the 10‐year‐old male patient presented with Class II malocclusion characterized by proclined incisors and bimaxillary crowding, along with severe traumatic injury to the maxillary left central incisor resulting in crown fracture. This case clearly illustrates the challenge of managing a traumatically injured tooth that undergoes early endodontic intervention but ultimately fails, leading to internal resorption and tooth loss. During treatment planning, options such as maintaining the space with a removable appliance while waiting for implant placement after growth completion or using temporary prosthetic replacements were considered. However, these approaches are limited in growing patients due to continuous changes in the alveolar bone and craniofacial structures. In contrast, autogenous tooth transplantation offers significant biological advantages by preserving a vital periodontal ligament, maintaining alveolar bone volume, supporting natural craniofacial development, and ensuring long‐term functional and esthetic outcomes. Consequently, it was selected as the optimal solution for this patient.

When a maxillary incisor becomes non‐restorable, selecting an appropriate and long‐lasting replacement option poses a major therapeutic challenge (Lee 2002; Gugnani et al. 2017). Implant placement is generally unsuitable in growing patients due to the risk of compromising the developing relationship between the teeth and alveolar bone (Kafourou et al. 2017), whereas fixed prostheses such as dental bridges may damage sound adjacent teeth and periodontal tissues (Palmer 2010). Consequently, autogenous tooth transplantation is considered a physiologic treatment option capable of preserving periodontal structures and supporting future alveolar and jaw development (Park et al. 2010; Aslan et al. 2010). Numerous studies have reported high success rates for this technique, especially when premolars are used to replace incisors, with long‐term success ranging from 80% to 100% (Andreasen et al. 1990; Czochrowska et al. 2002; Mendoza‐Mendoza et al. 2012; Stange et al. 2016). A key factor determining success is the survival and regenerative potential of the periodontal ligament. When viable periodontal ligament cells remain intact and establish proper contact with the recipient socket, fibroblasts can regenerate periodontal tissues and alveolar bone, maintaining the vitality of the transplanted tooth and enabling physiologic tooth mobility (Almpani et al. 2015; Tsukiboshi 2002; Ohshima et al. 2024). Extracorporeal time is therefore critical, if the donor tooth is transplanted promptly, the likelihood of survival and periodontal healing increases significantly (Almpani et al. 2015). Healing progresses through multiple stages, beginning with blood clot formation and fibrin network stabilization, followed by reattachment and reorganization of periodontal fibers into the alveolar socket within 2–8 weeks (Tsukiboshi 2002; Montesinos et al. 2025). Recent molecular biology studies have demonstrated significant gene expression changes within the periodontal ligament—particularly around day 28—which play an essential role in healing and long‐term success (Ohshima et al. 2024). The decision to use tooth 45 as the donor tooth in this case was supported by existing literature and by orthodontic considerations. According to published guidelines, mandibular premolars are ideal donor candidates for replacing maxillary anterior teeth due to similarities in cervical dimensions and root length, which help restore the biologic width and achieve favorable gingival esthetics (Czochrowska and Plakwicz 2020). Although the mandibular first premolar is traditionally preferred, contemporary clinical evidence and morphologic analyses indicate that the mandibular second premolar is equally suitable. A long‐term case report by Piroozmand et al. (2018) demonstrated stable outcomes using a mandibular second premolar to replace a traumatized maxillary central incisor, after 13 years of follow‐up, the transplanted tooth remained functional, free of pathologic resorption, and highly esthetic after reshaping. Additionally, the application of three‐dimensional imaging technologies, as proposed by Ambrósio et al. (2022) facilitates confirmation of root compatibility between the mandibular second premolar and the maxillary central incisor socket, thereby improving prognostic predictability. Compared with other donor sources such as supernumerary teeth or canines—which are often limited by root morphology or their role in guiding occlusion—premolars offer superior advantages, including survival rates exceeding 90% and long‐term esthetic stability. These findings are well supported by the classic works of Andreasen et al. (1990) and Czochrowska et al. (2002). In our clinical case, tooth 45 was positioned lingually and would not have been favorable to retain in the orthodontic treatment plan. It was therefore indicated for extraction as part of the planned premolar extraction strategy to address crowding. Utilizing tooth 45 both as a strategic extraction and as a donor tooth for the replacement of tooth 21 provided an optimal balance between morphologic suitability and comprehensive treatment objectives. In the present report, transplantation outcome was evaluated based on previously published criteria distinguishing survival and success. Survival was defined as the continued presence of the transplanted tooth in the oral cavity at follow‐up. In contrast, success required stricter clinical and radiographic criteria, including the absence of pain or pathologic mobility, probing depths ≤ 3 mm, maintenance of normal masticatory function, a clearly visible periodontal ligament space on radiographs, and the absence of progressive root resorption or periapical pathology (Andreasen et al. 1990; Czochrowska et al. 2002). Mild gingival redness around the transplanted tooth was observed at the completion of orthodontic treatment. This finding may be related to the previous chronic periapical lesion and sinus tract associated with tooth 21 prior to extraction, which may have affected the local soft tissue condition. In addition, the difference in cervical morphology between a transplanted premolar and a natural maxillary central incisor may influence the adaptation of the marginal gingival tissues. Importantly, no periodontal attachment loss or radiographic bone loss was detected during long‐term follow‐up, suggesting that this represented localized gingival inflammation rather than a periodontal complication of the transplanted tooth. The 7‐year follow‐up demonstrated stable function of the transplanted tooth and healthy periodontal healing, reinforcing evidence that the mandibular second premolar is an effective donor option for replacing maxillary incisors in growing patients.

An important consideration is that in teeth with incompletely developed roots, the pulp retains the capacity for revascularization, whereas in fully developed teeth, early endodontic treatment after transplantation is typically recommended to prevent complications (Tsukiboshi 2002; Tan et al. 2023). The stage of root development of the donor tooth is therefore a critical determinant of pulpal healing after autogenous tooth transplantation. Teeth with incomplete root formation and wide open apices generally demonstrate higher rates of pulp revascularization, whereas teeth with more advanced root development have a lower likelihood of maintaining pulp vitality (Andreasen et al. 1990; Czochrowska et al. 2002; Tan et al. 2023). In the present case, the donor tooth corresponded approximately to stage IV of Cvek's classification, showing nearly complete root formation with a relatively small apical opening, which may explain the absence of continued root development and the early occurrence of pulpal necrosis despite satisfactory periodontal healing. However, the transplanted tooth had an open apex and was intentionally preserved with the initial aim of allowing continued root development and natural apex closure. After a period of follow‐up, when no further apical development was observed and signs of pulpal necrosis became evident, endodontic treatment was initiated. This approach reflects a biologically oriented treatment philosophy aimed at maximizing the preservation of vital tissues, reducing the risk of complications, and improving long‐term prognosis (Tsukiboshi 2002). A key issue in this clinical case was the presence of Class II malocclusion with proclined incisors and crowding, a condition commonly seen in pre‐adolescent patients. Orthodontic treatment is the standard method to improve function and esthetics in such scenarios. The decision to extract four teeth (two in each arch) was appropriate in the context of moderate‐to‐severe crowding, as it provided the necessary space for alignment, reduced incisor protrusion, and improved the overall occlusal relationship (Vaden et al. 2018). An alternative treatment option could involve extraction of tooth 21 followed by orthodontic space closure with substitution of teeth 22 and 23. However, in patients with Class II malocclusion and maxillary crowding, such mechanics would require coordinated movement of multiple anterior teeth, increasing anchorage demands and potentially compromising posterior occlusal relationships. In addition, substitution of a lateral incisor or canine for a maxillary central incisor often requires significant reshaping to achieve acceptable esthetics (Czochrowska et al. 2003). In contrast, autotransplantation allows tooth replacement at the original site, preserving the dental arch structure, maintaining the physiological periodontal ligament, and facilitating better bilateral occlusal development. Therefore, in this case, autotransplantation represents a more favorable option in terms of biomechanics, esthetics, and long‐term prognosis (Akhlef et al. 2025). Notably, the unique aspect of this report lies in the reversal of the conventional treatment sequence and the choice of stabilization method. Whereas traditional protocols typically involve performing the transplantation first and delaying orthodontic mechanics (3–6 months) to allow soft tissue healing (Piroozmand et al. 2018; Czochrowska and Plakwicz 2020; Ambrósio et al. 2022), fixed appliances were bonded and preliminary alignment was performed prior to surgery in this case. This strategy eliminated the “waiting period” entirely and shortened the overall treatment duration compared with standard approaches. However, this treatment sequence should be applied with caution. Fixed orthodontic appliances may increase plaque accumulation and gingival inflammation if oral hygiene is not well controlled, potentially affecting postoperative healing (Verrusio et al. 2018; Zachrisson and Zachrisson 1972). In addition, excessive orthodontic tooth movement, particularly incisor proclination, may reduce the thickness of the labial alveolar bone and predispose to dehiscence (Handelman 1996; Guo et al. 2021), which could compromise the stability of the recipient site. Therefore, pre‐transplant orthodontic treatment may be more appropriate in carefully selected cases, such as patients with significant crowding requiring premolar extraction and well‐defined space conditions, while it may be less suitable in patients with poor oral hygiene, active periodontal inflammation, or insufficient alveolar bone support. Equally important, whereas most previous case reports have used semi‐rigid composite splints or simple stainless steel wires (Chiang et al. 2020; Ferreira et al. 2015; Hariri and Alzoubi 2019), a 0.016 × 0.022‐inch Nickel‐Titanium rectangular archwire was employed as the stabilization method. The use of a rectangular wire within a full‐arch fixed appliance system provided superior three‐dimensional control, allowing immediate torque and axial correction of the transplanted tooth and preventing rotational displacement commonly associated with round‐wire splints. Furthermore, the superelastic properties of Nickel‐Titanium created a “flexible yet stable” anchorage framework that allowed minimal physiologic mobility while maintaining adequate stabilization (Berthold et al. 2009; Kwan et al. 2012). Flexible stabilization has been recommended to facilitate periodontal ligament healing and reduce the risk of replacement resorption (ankylosis) associated with rigid fixation (Day et al. 2020).

Autogenous tooth transplantation, when combined with orthodontic treatment, offers several advantages over other replacement options such as dental implants. Unlike implants, which osseointegrate rigidly into the bone, transplanted teeth can physiologically adapt to jaw growth, maintain masticatory function, preserve alveolar vitality, and stimulate the continuous development of the alveolar bone, periodontal ligament, and gingival tissues at the recipient site. Moreover, the transplanted tooth can be moved orthodontically to achieve an ideal occlusion (Park et al. 2010; Aslan et al. 2010). This not only preserves function, the alveolar ridge, and soft‐tissue architecture, but also ensures long‐term esthetics throughout growth. From these considerations, it is evident that the combination of orthodontic treatment and autogenous tooth transplantation is not merely a tooth‐replacement method, but rather a comprehensive treatment strategy that optimizes function, esthetics, and long‐term stability in growing patients. The clinical case presented provides rare evidence supporting the use of pre‐transplant orthodontic treatment in combination with autotransplantation of a mandibular premolar to replace a maxillary incisor. After 7 years of treatment and follow‐up, the results showed that the transplanted tooth maintained stable function, achieved satisfactory esthetics, and contributed to harmonious arch and jaw development. This case expands the clinical indications for autogenous tooth transplantation combined with orthodontic treatment in managing complex anterior tooth loss in young patients with crowding and malocclusion.

Despite the favorable outcome observed in this case, several limitations should be acknowledged. As a single case report, the findings have limited generalizability and may be influenced by case selection. In addition, the donor tooth presented with nearly complete root development and ultimately required endodontic treatment, indicating that pulp survival in similar cases may vary. Furthermore, three‐dimensional imaging was not used to quantitatively evaluate alveolar bone changes, and although the follow‐up period of 5–7 years demonstrated stable outcomes, it may still be insufficient to completely exclude very late complications such as replacement root resorption. Therefore, the present report should be interpreted primarily as evidence of clinical feasibility under selected conditions. The present scenario underscores the importance of comprehensive evaluation when selecting replacement options for growing patients. The combination of autogenous tooth transplantation and comprehensive orthodontic therapy may represent a biologically favorable treatment option in carefully selected cases for the rehabilitation of complex anterior tooth loss.

This clinical report illustrates a complex scenario in a young patient presenting with Class II malocclusion, bimaxillary crowding, and a maxillary left central incisor affected by post‐endodontic periapical pathology. Autotransplantation of the mandibular right second premolar to replace the maxillary left central incisor, combined with comprehensive orthodontic treatment, demonstrated both feasibility and long‐term effectiveness. Bonding brackets immediately after transplantation provided stabilization and early guidance of the transplanted tooth, contributing to reduced overall treatment time and stable functional and esthetic outcomes. The rarity of using a mandibular premolar to replace a maxillary incisor adds valuable evidence supporting the expanded indications of autogenous tooth transplantation and offers important clinical insights for managing complex anterior tooth loss in growing patients.

## Author Contributions

Conceptualization: Nga Thi Nguyen, Nga Minh Truong, and Hanh Thi‐My Tran. Data curation: Nga Thi Nguyen. Formal analysis: Nga Thi Nguyen and Nga Minh Truong. Investigation: Nga Thi Nguyen. Methodology: Nga Thi Nguyen and Nga Minh Truong. Project administration: Nga Thi Nguyen and Nga Minh Truong. Resources: Nga Minh Truong. Supervision: Nga Thi Nguyen and Nga Minh Truong. Validation: Nga Thi Nguyen, Nga Minh Truong, and Hanh Thi‐My Tran. Visualization: Nga Thi Nguyen, Nga Minh Truong, and Hanh Thi‐My Tran. Writing – original draft: Nga Minh Truong and Hanh Thi‐My Tran. Writing – review and editing: Nga Minh Truong and Hanh Thi‐My Tran.

## Funding

The authors have nothing to report.

## Ethics Statement

Written informed consent to participate was obtained from the patient's parent/legal guardian.

## Consent

Written informed consent was obtained from the patient's parent/legal guardian for publication of this case report and any accompanying clinical images. A copy of the written consent is available for review by the journal's Editor upon reasonable request.

## Conflicts of Interest

The authors declare no conflicts of interest.

## Data Availability

All data generated or analyzed during this case report are included in this published article. Additional details (if requested) are available from the corresponding author on reasonable request.
